# Cocktail of Hyaluronic Acid and Human Amniotic Mesenchymal Cells Effectively Repairs Cartilage Injuries in Sodium Iodoacetate-Induced Osteoarthritis Rats

**DOI:** 10.3389/fbioe.2020.00087

**Published:** 2020-03-06

**Authors:** Ai-Tong Wang, Qing-Fang Zhang, Nuo-Xin Wang, Chang-Yin Yu, Ru-Ming Liu, Yi Luo, Yu-Jie Zhao, Jian-Hui Xiao

**Affiliations:** ^1^Zunyi Municipal Key Laboratory of Medicinal Biotechnology, Affiliated Hospital of Zunyi Medical University, Zunyi, China; ^2^Center for Translational Medicine, Affiliated Hospital of Zunyi Medical University, Zunyi, China; ^3^Department of Neurology, Affiliated Hospital of Zunyi Medical University, Zunyi, China; ^4^Department of Laboratory Medicine, Affiliated Hospital of Zunyi Medical University, Zunyi, China

**Keywords:** osteoarthritis, human amniotic mesenchymal cells, hyaluronic acid, chondrogenic differentiation, cartilage repair

## Abstract

Osteoarthritis (OA) is one of the most common refractory degenerative articular cartilage diseases. Human amniotic mesenchymal cells (hAMSCs) have emerged as a promising stem cell source for cartilage repair, and hyaluronic acid (HA) has proven to be a versatile regulator for stem cell transplantation. Herein, an effective and straightforward intra-articular injection therapy using a cocktail of hAMSCs and HA was developed to treat knee OA in a rat model. The injured cartilage was remarkably regenerated, yielding results comparable to normal cartilage levels after 56 days of treatment. Both hAMSCs and HA were indispensable organic components in this therapy, in which HA could synergistically enhance the effects of hAMSCs on cartilage repair. The regenerative mechanism was attributed to the fact that the addition of HA comprehensively enhances the activities of hAMSCs, including chondrogenic differentiation, proliferation, colonization, and regenerative modulation. This cocktail paves a new avenue for injection therapy to treat OA, holding the potential to realize rapid clinical translation.

## Introduction

Osteoarthritis (OA), a late-stage joint cartilage injury, affects more than a quarter of the adult population around the world, including more than 50 million people in the United States and more than 100 million people in China (Wang and Cao, 2014; Chen et al., 2017). It is characterized by the gradual loss of articular cartilage accompanied by a series of pathological changes, such as hyperplasia of the synovium, osteosclerosis of the subchondral bone, and osteophytosis (Loeser et al., 2012; Kim et al., 2014; Wang and Cao, 2014). Its clinical manifestations include joint pain, joint deformity, and limited normal joint activities. Thus, OA will seriously lower affected patients’ quality of life and even lead to disability; treatment for OA, therefore, is urgent and under intensive investigation (Huang et al., 2016).

As a kind of elastic structure connecting bones, the articular cartilage has special structural and compositional characteristics. It does not contain blood vessels, nerves, or lymphoid tissues to supply nutrients, and its nutrients supply relies mainly on synovial penetration; also, mature articular chondrocytes are mainly hyaline chondrocytes, with a limited proliferative and self-healing capacity (Gomoll and Minas, 2014). Therefore, the regeneration of articular cartilage after injury is challenging. Although routine treatments, such as subchondral drilling, microfracture, periosteum/perichondrium transplantation, and cartilage tissue grafting, relieve the symptoms of the patients to a certain extent, their long-term treatment efficacy is not satisfactory (Yang et al., 2012; Bark et al., 2014; Musumeci et al., 2014; Bhattacharjee et al., 2015). These routine treatments commonly only alleviate pain, ameliorate joint function, and minimize disability but do not regenerate cartilage. In addition, these treatments are conducted in a heavily invasive manner with complicated procedures, which pose great risks for serious patient suffering and complications during or after the surgery, and produce burdening workloads for medical staff. Accordingly, there is an enormous clinical need for a more effective and simple treatment of OA.

In recent years, intra-articular injection of somatic cells with simple and minimally invasive procedures has proven to be a promising pathway (Lopa et al., 2018). Currently, two types of cells, chondrocytes and adult stem cells (ASCs), are mainly used for injection. Because of reliance on invasive acquisition and the limited sources, chondrocyte-based therapy struggles to meet the needs of scientific research and clinics. Thus, ASCs are considered to be more ideal sources of seed cells for cartilage rehabilitation. Bone marrow-derived mesenchymal stem cells (BMSCs) are currently the most widely used ASCs for cartilage repair (Huang et al., 2013). BMSCs have a strong proliferative and differential capacity. They can differentiate into chondrocytes as well as into various other cell types, such as osteoblasts and vascular cells, under suitable conditions. However, the acquisition of BMSCs is still invasive, which can cause secondary damage and suffering to patients. Additionally, BMSCs’ quality largely fluctuates based on a patient’s age and health status, which complicates the clinical application. Therefore, a more favorable stem cell is highly sought after for the application of injection therapy of cartilage repair.

Currently, human amniotic mesenchymal stem cells (hAMSCs) show great promise due to their outstanding properties (Muiños-López et al., 2017). hAMSCs are an emerging kind of stem cell isolated from human placenta (a detached and discarded organ). hAMSCs are not only obtained non-invasively but also much more stable in quality than ASCs, abundant and accessible in sources, and pose few ethical issues. They originate from the extra-embryonic mesoderm of the primitive streak, suggesting high cellular activity and differentiation capacity; they are capable of differentiating into chondrocytes in culture; and they have been identified to have similar phenotypes and characteristics to BMSCs, but with a stronger amplification and differentiation ability (Alviano et al., 2007; Ilancheran et al., 2007; Wei et al., 2009; Liu et al., 2016). They also exhibit non-immunogenicity and immunoregulatory activity, as well as an anti-fibrotic effect (Tsuno et al., 2012; Liu et al., 2016; Navas et al., 2018). Furthermore, they belong to the lineage of the mesoderm, the same germ layer as chondrocytes, and additionally, their resident environment, the amnion, is also a tissue without blood vessels, nerves, or lymphoid tissue, quite similar to the joint cartilage. Recently, *in vitro* models have demonstrated that hAMSCs are favorable sources of cartilage repair (Muiños-López et al., 2017; Topoluk et al., 2017, 2018). However, to date, reports on hAMSCs-based cartilage repair *in vivo* are rare, and to our knowledge, studies on the injection treatment of cartilage damage *in vivo* by hAMSCs have not been reported (Rameshbabu et al., 2016).

Hyaluronic acid (HA) is an unbranched non-sulfated glycosaminoglycan with excellent biocompatibility and low immunogenicity (Garg and Hales, 2004; Kurisawa et al., 2005; Andre, 2008). As a major component of the extracellular matrix (ECM) and the stem cell niche, HA takes part in diverse biological processes such as cell proliferation, differentiation, and migration (Lee and Spicer, 2000). In addition, HA has been found to be important in the composition of cartilage tissue and articular synovial fluid in joints (Asari et al., 1994). HA has been widely used for differentiating ASCs into chondrocytes and repairing articular cartilage damage (Toh et al., 2010). Besides, HA injections, also known as viscosupplements, have been approved by the United States Food and Drug Administration (FDA) for the treatment of knee OA. Considering these factors, we hypothesize that the addition of HA will further enhance the therapeutic effect of hAMSCs on OA.

In the present study, the efficacy of the injection treatment of knee OA by the combination of HA and hAMSCs in a rat model was investigated for the first time. We also explored the healing mechanism by (i) evaluating the *in vitro* chondrogenic differentiation effect of HA on hAMSCs, (ii) performing histological and magnetic resonance imaging (MRI) examinations, and (iii) analyzing the secretion of the regeneration-modulatory cytokines. Our data indicate that the combined treatment could dramatically regenerate damaged cartilage in rat OA models, whose mechanism encompasses enhanced chondrogenic differentiation, proliferation, enhanced colonization and incorporation of hAMSCs into damaged cartilage, and enhanced modulation of cytokine secretion to optimize the regenerative microenvironment, by the addition of HA.

## Materials and Methods

### Cell Isolation and Culture

The use of human placentas was approved by the Ethical Committee of the Affiliated Hospital of Zunyi Medical University. Human term placentas were collected from healthy volunteer donors undergoing caesarean sections after they had provided written informed consent in accordance with the Declaration of Helsinki. The placentas were then washed immediately thrice with phosphate-buffered saline (PBS) containing 200 U/mL penicillin and 100 μg/mL streptomycin. Isolation of hAMSCs was performed by a modified two-step enzyme digestion method as described previously (Liu et al., 2016). Briefly, the amniotic membrane was mechanically peeled off from the chorion of the placenta, washed with PBS, and then cut into pieces. The resulting minced amnion was digested with 0.05% trypsin (Gibco, New York, United States) in low-glucose Dulbecco’s Modified Eagle’s Medium (LG-DMEM; Gibco, New York, United States) and incubated at 37°C in an incubation box while being stirred at 180 rpm for 50 min in a water bath shaker (SHZ-A, Boxun, Shanghai, China). The mixture was then poured over a 300 mesh filters to remove the dispersed amnion epithelial cells from the tissue pieces. The digestion and the filtration were repeated several times until no further amniotic epithelial cells could be obtained. Subsequently, hAMSCs were isolated from remnant amniotic tissue pieces. The tissue pieces were placed in LG-DMEM with collagenase (0.75 mg/mL; Sigma, Saint Louis, United States) and DNase I (0.075 mg/mL; BioBasic, Toronto, Canada) and were incubated at 37°C, while being stirred at 200 rpm for 60 min in a water bath shaker (SHZ-A, Boxun, Shanghai, China). The dispersed hAMSCs were collected by filtration through 300 mesh filters and centrifugation. The harvested hAMSCs were cultured in LG-DMEM complete medium at a density of 20,000 cells/cm^2^, supplemented with 10% (v/v) heat-inactivated fetal bovine serum (FBS; Gibco, New York, United States), 1% (v/v) GlutaMAX (Gibco, New York, United States), 1% (v/v) non-essential amino acids (NEAA; Gibco, New York, United States), 55 μM β-mercaptoethanol (Gibco, New York, United States), 10 ng/mL basic fibroblast growth factor (bFGF; Sigma, Saint Louis, United States), and 1% (v/v) antibiotics. The cultures were maintained at 37°C in an incubator with an atmosphere consisting of 5% CO_2_, 95% air, and 100% relative humidity. When 80% confluence was reached, the cells were passaged into T25 bottles at a density of 20,000 cells/cm^2^. Cells at passage 3 (P3) were used in this study. In our study, we used placentas from three donors to carry out our experiments.

### Phenotypic Identification of hAMSCs

For the flow cytometry analysis, the hAMSCs at P3 were harvested and stained with allophycocyanin (APC)-conjugated antibody against CD73, fluorescein isothiocyanate (FITC)-conjugated antibody against CD90, PerCP-Cyanine 5.5 (PerCP-Cy5.5)-conjugated antibody against CD105, and phycoerythrin (PE)-conjugated antibodies against CD11b, CD19, CD34, CD45, and HLA-DR (an MHC class II antigen). All antibodies were purchased from BD Pharmingen (New Jersey, United States). Here non-immune isotype controls including IgG2b-PE, IgG2a-PE, and IgG1-FITC/PerCP-Cy5.5/APC/PE, from the same company, were employed in this study. Cell fluorescence was examined by flow cytometry in a FACS Calibur instrument (BD, New Jersey, United States).

For the immunocytochemistry analysis, P3 hAMSCs in six-well chambers were fixed in 4% paraformaldehyde for 30 min. After washing with D-PBS, the cells were permeabilized with 0.5% (v/v) Triton X-100 for 10 min at room temperature. Following three more washes with D-PBS, non-specific binding was blocked with 10% (v/v) normal goat serum for 30 min at room temperature in the dark. The cells were incubated overnight at 4°C with primary antibodies against vimentin (1:100; Sigma, United States) or human cytokeratin 19 (CK19, 1:100; Gene Tech, Shanghai, China) in D-PBS containing 0.2% Triton X-100. The cells incubated with PBS were used as the control. The cells were then washed and incubated for 30 min with horseradish peroxidase-conjugated goat anti-mouse IgG secondary antibody. After washing with PBS, the cells were stained using a DAB kit (ZSGB-BIO, Beijing, China) and observed under the microscope (IX-71-S8F, Olympus, Japan). Nuclei were counterstained with hematoxylin.

### Chondrogenic Differentiation of hAMSCs

For evaluating the effect of HA on the chondrogenic differentiation of hAMSCs, P3 hAMSCs were seeded in a six-well plate at a density of 1 × 10^5^/mL, and the differential medium (high-glucose Dulbecco’s Modified Eagle’s Medium (HG-DMEM; Gibco, New York, United States) +10% FBS + 2 mM GlutaMAX + 1% NEAA + 55 μmol/L β-mercaptoethanol + 10 ng/L TGF-β3 (Peprotech, Rocky Hill, United States) +10^–7^ mol/L Dex (Sigma, Saint Louis, United States) +50 mg/L VitC (Sigma, Saint Louis, United States) supplemented with HA (0.05 mg/mL, 300 kDa) was added to cells in logarithmic growth phase (denoted as CG group). The cells were observed or manipulated at the 7th, 14th, 21st, and 28th day after induction. Three kinds of other media were used in control groups, including NC group using differential medium without TGF-β3, Dex and VitC, PG group using differential medium only, and HA group using differential medium without TGF-β3, Dex, and VitC but supplemented with HA (0.05 mg/mL, 300 kDa). The medium was replaced every 3 days. The experiment was repeated three times.

### Monoiodoacetate-Induced Osteoarthritis

The procedure of animal trials was approved by the Ethical Committee of Affiliated Hospital of Zunyi Medical University. Male SD rats were housed in the central laboratory (Zunyi Medical University) and used at least 1 week after their arrival. The rats were fed with a standard laboratory diet and tap water *ad libitum*, and kept at 23 ± 1°C with a 12/12 h light/dark cycle (light at 7 a.m.). Rats were anaesthetized before cervical dislocation. All efforts were made to minimize animal suffering and to reduce the number of animals used. Unilateral osteoarthritis was induced by injection of monoiodoacetate (MIA; Sangon, Shanghai, China) into the knee joint according to the described method (Guingamp et al., 1997; Pomonis et al., 2005). In brief, on day 1, after rats were slightly anesthetized by 6% chloral hydrate, the left leg skin was sterilized with 75% ethyl alcohol and the knee was located by palpation; then, a 28-gauge needle was inserted vertically to penetrate the skin and turned distally for insertion into the articular cavity until a distinct loss of resistance was felt. 20 μg/μL of MIA in 100 μL saline was delivered into the left articular cavity. Control rats received 100 μL of saline solution (day 1) in the knee joint. Behavioral, biochemical, and imaging measures were performed at day 14 to confirm the successful establishment of the OA model.

### Treatment of Osteoarthritis

In this study, the treatment efficacy of HA group (HG), P3 hAMSCs transplantation group (HTG), and their combined transplantation group (CTG) was evaluated, while normal rats without OA induction (NG) and OA-induced rats left without any treatment (MG) were used as controls. The volume of injected liquid for all treatment groups was 100 μL. For the HG group, 100 μL LG-DMEM containing 0.05 mg/mL HA was used; for the HTG group, 100 μL of LG-DMEM containing 1 × 10^6^ cells was used; for the CTG group, 100 μL LG-DMEM containing both 0.05 mg/mL HA and 1 × 10^6^ cells was used. There were six rats in each group. Intra-articular injection treatment was conducted at days 14 and 42 twice after model establishment for all groups, and the efficacy was observed until day 70 (56 days after treatment). HE staining, toluidine blue staining, and immunohistochemistry were employed to comprehensively evaluate the treatment effect of the arthritis. To track hAMSCs in rats by magnetic resonance imaging (MRI), P3 hAMSCs in favorable growth status were washed twice with D-PBS, labeled for 18 h with superparamagnetic iron oxide (SPIO; BIoPAL, Guangzhou, China) containing 25 mg/mL Fe^3+^, and digested with 0.125% trypsin to prepare a cell suspension. Prussian blue staining was performed to calculate the labeling rate. After staining, five fields were observed randomly, and the labeling rate is the percentage of Prussian blue-positive cells to total cells. The labeled cells were then injected intra-articularly within 1 h.

### Magnetic Resonance Imaging

The MRI was performed using a 3.0 T large-scale clinical MRI scanner (GE Signa HD xt 3.0 T, United States). The rat body coil and the supine position tape were used to fix the rat so that the right posterior knee joint was located in the middle of the scanning coil. Scanning sequence: T2WI (TE/TR = 3040.0/92.0 ms), NEX = 2.0, FOV = 6.00 cm × 6.00 cm, GE, scanning direction: sagittal and transverse, scanning layer thickness: 1 mm, matrix: 256 × 256, bandwidth: 14.7 kHz. After the modeling was completed, rats from the normal group and the model group were randomly taken for MRI imaging; on days 21, 35, and 56 after treatment, 1–2 rats were randomly selected from each group (each containing six rats), weighed, and scanned by MRI after intraperitoneal injection of 6% chloral hydrate (0.5 mL/100 g).

## Elisa

After treatment administration of 4 and 8 weeks, experimental rats (six rats in each group at each time point) were all anesthetized with 6% chloral hydrate by intraperitoneal injection (0.5 mL/100 g). The blood was taken from the aorta abdominalis and centrifuged at 3,000 × *g* for 20 min to separate the plasma in the supernatant. The synovial fluid was aspirated from the knee joint cavity using a syringe. The cytokines TNF-α, IL-4, IL-6, IL-1b, IL-10, and osteoprotegerin (OPG) were detected separately by their ELISA kit according to the manufacturer’s instructions. The correlation of standard curves was tested by GraphPad software. The parameter settings were “Compute r for X versus every Y data set,” “Pearson correlation coefficients,” and “two-tailed *P* value.” All ELISA kits were purchased from BiosaMITE Co., Ltd. (Shanghai, China).

### Histological and Immunological Analysis of Cells and Tissue Sections

For GAG and collagen staining of cell cultures, monolayer cultures were harvested and fixed in 4% PFA. Sections were stained with toluidine blue (specific to matrix proteoglycans). Expression of collagen II was detected by immunohistochemistry staining with specific primary antibodies, biotinylated secondary antibodies, and the ABC method with diaminobenzidine (DAB) as the chromogen.

For histological and immunological staining of the tissue, harvested tissues were fixed by 4% PFA, then decalcified by 10% EDTA-2Na solution at room temperature for 28 days, embedded in paraffin, and sectioned into 5 μm-thick slices. The slices were fixed on glass slides, deparaffinized, and then stained with toluidine blue for GAG. Collagen II was labeled using an immunofluorescent method. Briefly, deparaffinized tissue sections were incubated with citrate buffer heated to 99°C for 25 min to retrieve antigens, followed by a 20-mincooling at room temperature. The slides were then incubated with 3% BSA in PBS for 60 min before being washed three times using PBS. The slides were incubated with FITC-conjugated mouse anti-human MAB1281 antibody (1:100; Millipore, Ontario, Canada) or PE-conjugated rabbit anti-human collagen II antibody (1:100; Abcam, Cambridge, United Kingdom) over night at 4°C. After they were washed with PBS thrice, the samples were treated with goat anti-mouse or anti-rabbit IgG secondary antibodies for 60 min at 37°C. The nuclei were co-stained with DAPI. Color images were captured under a fluorescent microscope (Leica, Germany). Histological sections from each defect were blindly scored by three independent researchers based on Histologic/Histochemical Grading System (HHGS), a previously established scoring system by Mankin et al. (1981).

### Quantitative Reverse Transcription PCR

The primers for *Col2a1* were synthesized by Shanghai Invitrogen Biotechnology Co., Ltd. The details of the primer are Forward: CAACACTGCCAACGTCCAGAT, Reverse: TCTTGC AGTGGTAGGTGATGTTCT. The reference gene used for PCR analysis was β*-actin* (Forward: TGGCACCCAGCACAATGAA, Reverse: CTAAGTCATAGTCCGCCTAGAAGCA). On the day 56 of treatment, the cartilage tissues were taken out, washed with D-PBS, lysed with Trizol reagent, and stored at −80°C till use. After RNA was extracted, its purity and concentration were measured by OD_260_/OD_280_ and OD_260_, respectively, and its integrity was validated by 0.8% agarose gel electrophoresis. cDNA was obtained by reverse transcription using PrimeScript^TM^ RT reagent Kit (Perfect Real Time) (TaKaRa, Dalian, China). Then real-time qPCR was performed using SYBR Premix Ex Tap^TM^ system (TaKaRa, Dalian, China). The result was analyzed by 2^–ΔΔCt^ method. The specificity of the products was verified by melting curve analysis and 1.8% agarose gel electrophoresis.

### Statistical Analysis

All tests were performed for at least three independent replicates. Data were given as mean ± standard deviation (sd). Statistical significance was assessed by a one-way ANOVA test for multiple groups comparison and the unpaired Student’s *t*-test between two groups by GraphPad software. The normal distribution of the data is verified by D’Agostino-Pearson omnibus normality test. Any *P*-value less than 0.05 was considered to be statistically significant.

## Results

### Characterization of hAMSCs

**FIGURE 1:**
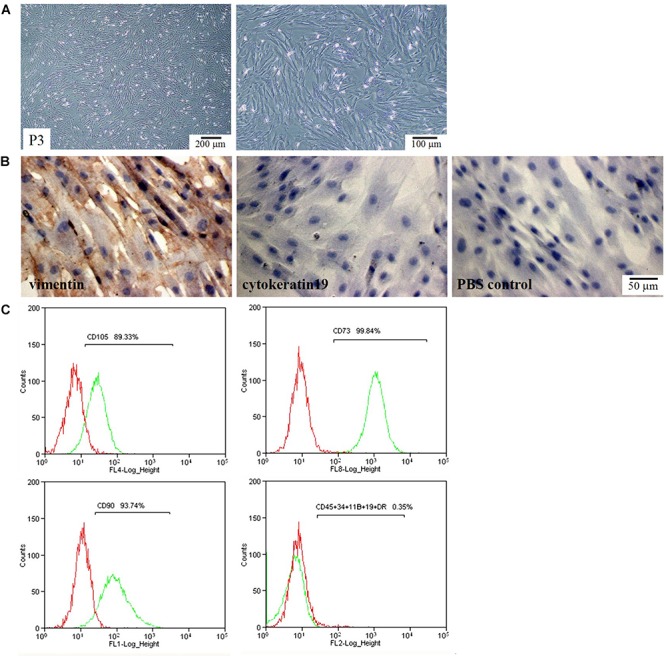
Morphological and phenotypic characteristics of hAMSCs. **(A)** Optical microscope images of hAMSCs. Left: 40×, Right: 100×. **(B)** The expression of vimentin and cytokeratin 19 (CK19). **(C)** Flow cytometric analysis of cell surface makers. Shown here are the sorted sub-populations that stained positively with CD105, CD73, and CD90, and negatively with CD45, CD34, CD11B, CD19, and HLA-DR. Red: Isotype control; Green: hAMSCs.

We first checked the morphology and phenotype of hAMSCs. hAMSCs exhibited a polymorphic fibroblast-like morphology (Figure 1A). They expressed mesenchymal cell marker vimentin, but did not express the epithelial marker CK19 (Figure 1B). Flow cytometry confirmed positive expression of the mesenchymal stem cell-specific markers CD90, CD73, and CD105. In contrast, the cells were negatively labeled by the hematopoietic stem cell markers CD34, CD45, CD11B, CD19, and HLA-DR (Figure 1C).

### Effects of HA on hAMSCs Differentiation Into Chondrocytes *in vitro*

**FIGURE 2:**
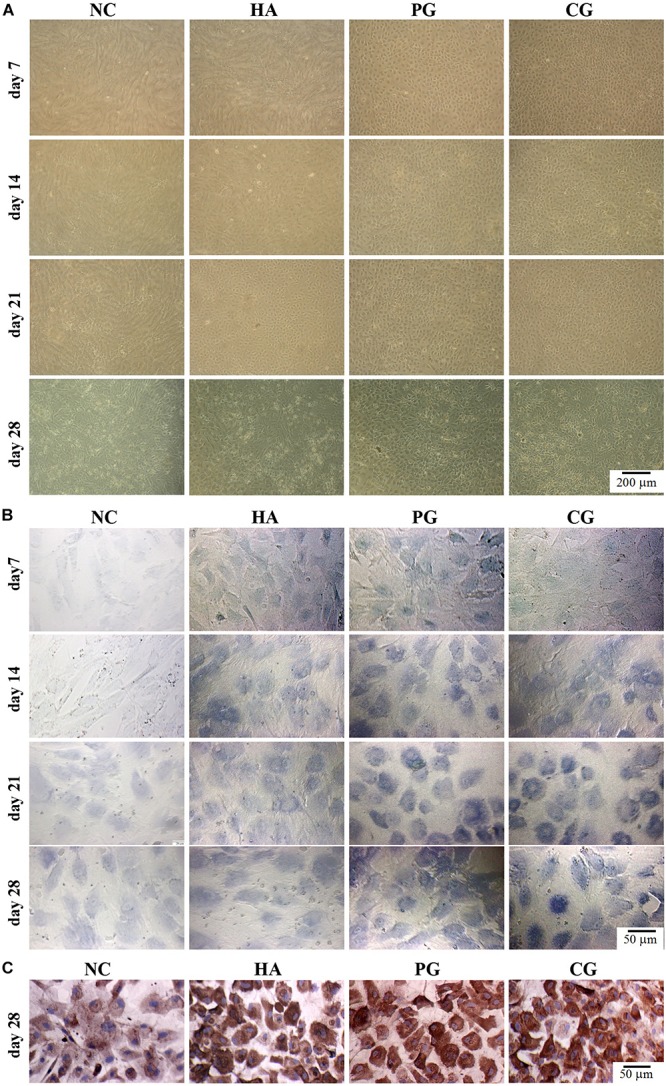
Visual observations of hAMSCs differentiating into chondrocytes *in vitro*. **(A)** Morphology. **(B)** Accumulation of glycosaminoglycan by toluidine blue staining. **(C)** Expression of collagen II by immunohistochemical staining. NC, HG-DMEM; PG, chondrogenic differentiation medium; HA, HG-DMEM + 0.05 mg/mL HA; CG, 0.05 mg/mL HA + chondrogenic differentiation medium.

We then evaluated the effects of HA on chondro-differentiation of hAMSCs. As shown in Figure 2A and Supplementary Figure S1A, at day 7 of induction, cells in the NC and HA group were still in a normal proliferative stage (elongated fusiform-like shapes), while those in PG and CG group were in a chondrogenic differential stage (paving stone-like shapes), and the chondrogenic differentiation in CG group was even quicker; cells in the HA group partially entered the chondrogenic differential stage at day 14 and almost completed the differentiation at day 21 (Alves da Silva et al., 2015). As shown in Figures 2B,C and Supplementary Figures S1B,C, there were almost no vision variations for the GAG accumulation and collagen II expression in cells between PG and CG groups at the induction duration. These overall results indicatively suggest that HA alone possesses a similar function to routine chondro-inductive chemicals on the chondrogenic differentiation of hAMSCs and a promotive effect on the chondrogenic differentiation of hAMSCs by chondro-inductive chemicals to some extent.

### Establishment of the Rat Osteoarthritis Model

To evaluate the treatment efficacy of the HA and hAMSCs combination, we established the osteoarthritis model in the knee joints of rats. After 2 weeks of model induction, the rats showed claudication and joint swelling (Supplementary Figure S2). After explantation, the normal group’s (NG) joint surface was smooth, while the model group’s (MG) was swollen with joint effusion and the articular cartilage was partially damaged (Supplementary Figure S3A). As shown in Supplementary Figure S3B, HE staining illustrated that the cells in the normal group were arranged neatly with clusters each containing 2–8 cells and that the tidal line was clear; on the other hand, the chondrocytes in the model group were disorderly arranged, the chondrocytes were reduced with a decreased degree of phagocytosis and a blurred tidal line, and the cartilage layer became thin. Toluidine blue staining showed that the normal group was heavily stained, but the model group was lightly stained. Immunohistochemistry demonstrated the positive expression in the normal group and weak positive expression in the model group. MRI showed normal signals in the normal group, viz., except for the high T2 signal of the joint fluid in a white color, the cartilage showed a medium T2 signal in a gray color; the joints of the model group (MG) mainly showed a high signal indicating the existence of the inflammation and cartilage damage in the joint (Supplementary Figure S3C). These results confirmed that the cartilage injury model was successfully established by MIA treatment.

### Evaluation of Treatment Efficacy by MRI

To evaluate the treatment efficacy by MRI, hAMSCs were labeled with SPIO before injection (Supplementary Figure S4). Prussian blue staining revealed the labeling rate could reach up to 96.5%.

**FIGURE 3:**
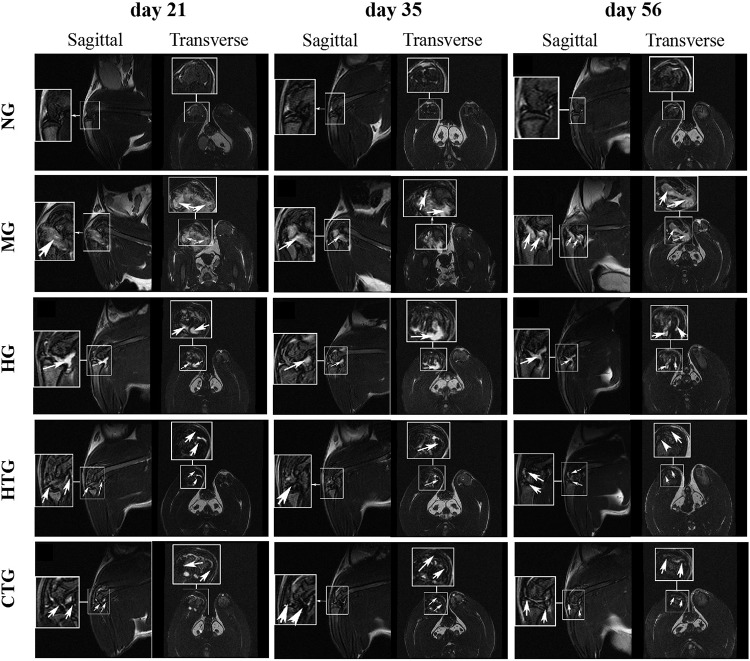
MRI imaging of the knee joints of rat OA models after treatment. NG, LG-DMEM; MG, Model group; HG, 0.05 mg/mL HA; HTG, hAMSCs 1×10^6^; CTG, HA (0.05 mg/mL) + hAMSCs (1×10^6^). In the MG and HG groups, the arrows indicate the high signal of MRI showing the cartilage injury/inflammation; in the HTG and CTG groups, the arrows indicates the low signal of MRI showing the presence of hAMSCs-derived cells labeled by SPIO. 1–2 rats randomly selected from each group of six rats were used for MRI analysis.

At days 21, 35, and 56 after treatment, MRI was performed on each group. The articular cartilage of the model group showed a much higher T2 signal than the normal group, indicating that there was severe inflammation and cartilage damage in the joints (Figure 3). Although lower than at days 21 and 35, there were still high T2 signals in the HA-treated group (HG) and the hAMSCs-treated group (HTG) at day 56. In contrast, the combination treatment group by HA and hAMSCs (CTG) showed mainly low T2 signals, and the signal intensity decreased with treatment time (Figure 3), illustrating an efficient inflammation suppression and therapeutic outcome. In addition, as shown by the fraction of the lowest T2 signal in MRI imaging in a dark color, the SPIO-labeled hAMSCs were mainly distributed in the cartilage injury tissues in the joint cavity, both in the HTG and the CTG group, indicating that some hAMSCs survived and had migrated to the cartilage injury site in these groups (Figure 3).

### Gross Changes of Cartilage Tissues

**FIGURE 4:**
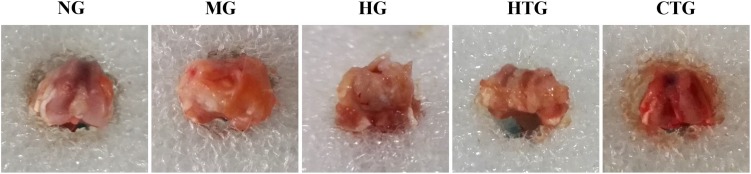
Gross observations of knee joint cartilage repair of rat OA models after 56 days of treatment. NG, LG-DMEM; MG, Model group; HG, 0.05 mg/mL HA; HTG, hAMSCs 1×10^6^; CTG, HA (0.05 mg/mL) + hAMSCs (1×10^6^). This figure shows the representative rat joints of each group containing six rats.

We observed the gross morphology of obtained cartilage tissues. After 56 days of treatment, it could be noticed that there were joint effusion, synovial hyperplasia, and severe cartilage surface damage in the MG group (Figure 4). Although cartilage surfaces were still rough, irregular, and erosive, and effusion still existed, in both the HG and HTG groups, there were small portions of cartilage regeneration that could be observed. In the CTG group, compellingly, we could see an almost-intact cartilage surface without joint effusion and synovial swelling. Consequently, HG alone and HTG alone both exhibited positive but limited repair effects, while the CTG showed obvious repair effects on the damaged cartilage (Figure 4).

### Pathological Changes of Cartilage Tissues

**FIGURE 5:**
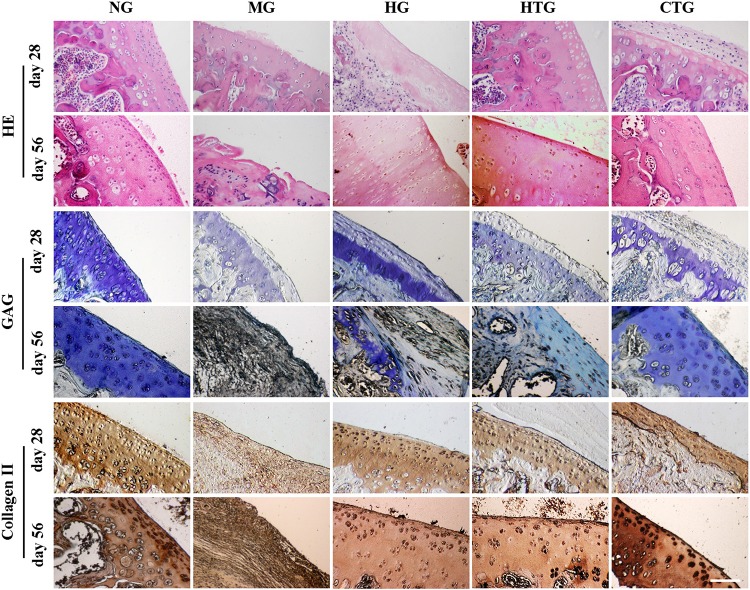
Pathological changes of knee joint cartilage repair of rat OA models after treatment. **Top:** HE, **Middle:** Tolune blue for GAG, **Bottom:** Immunohistological staining for Collagen II. NG, LG-DMEM; MG, Model group; HG, 0.05 mg/mL HA; HTG, hAMSCs 1×10^6^; CTG, HA (0.05 mg/mL) + hAMSCs (1×10^6^). Scale bar: 50 μm. This figure shows the representative tissue sections of each group containing six rats.

We then examined the pathological changes by tissue sections. After 28 days, HE staining showed that the cartilage surface of the MG group got destroyed, chondrocytes on the surface died, and the degree of phagocytosis faded (Figure 5). The cartilages in the HG group and the HTG group were slightly better than those of the model group, but chondrocyte reduction and a decrease of the phagocytosis degree also occurred. A thin layer of new cartilage-like cells with lighter staining can be observed on the basis of the original chondrocyte layer in the CTG group. After 56 days, HE staining showed that the cartilage layer of the MG group was severely damaged, and necrotic and disintegrating cells turned up, where the lesion had reached the subchondral bone and the tidal line disappeared. The cartilage layer of the HG and HTG groups was extremely thin, and a small number of chondrocytes in these two groups were observed. On the contrary, the joint in the CTG group showed remarkable filling of cartilage tissues in comparison to the other groups; the ECM of the repaired tissue was stained homogeneously with HE, integrating into the generated new cartilage (Figure 5). These results indicated slight regeneration in HG and HTG groups, but considerable regeneration in the CTG group.

Toluene blue staining for 28 days revealed that a much lighter staining occurred in the MG group, and the staining recovered in the HG, HTG, and CTG groups, in which the CTG group seemed the darkest (Figure 5). After 56 days, toluidine blue staining for the MG group was lost, and the HG and HTG groups were lightly stained, while the CTG group was heavily stained almost as dark as the NG group (Figure 5). These results indicated the slight GAG expression in the HG and HTG groups, but large amounts of GAG expression in the CTG group.

After 28 days, immunohistochemistry indicated that the expression of collagen II was weak in the MG group, and better positive expression was observed in the HG, HTG, and CTG groups. After 56 days, we could see almost negative expression of collagen II in the MG group, strong positive expression in the HG, HTG and CTG groups, in which CTG group showed a quite parallel collagen II expression to the NG group (Figure 5). These results indicated that positive collagen II expression in the HG, HTG, and CTG groups, in which the CTG group was the strongest.

**FIGURE 6:**
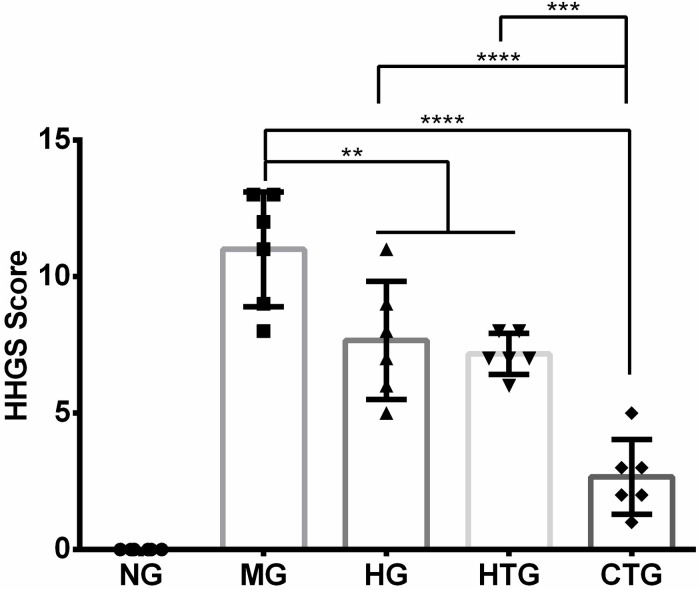
HHGS scoring of the cartilage after 56 days of treatment. NG, LG-DMEM; MG, Model group; HG, 0.05 mg/mL HA; HTG, hAMSCs 1×10^6^; CTG, HA (0.05 mg/mL) + hAMSCs (1×10^6^). The data are expressed as mean ± sd (*n* = 6), **P < 0.01, ***P < 0.001, ****P < 0.0001.

The quality of cartilage repairing after 56 days of treatment was evaluated by the HHGS method. The HHGS scores are 0 for NG, 11.00 ± 2.10 for MG, 7.67 ± 2.16 for HG, 7.17 ± 0.75 for HTG, and 2.67 ± 0.37 for CTG (Figure 6). There are no significant differences in HHGS scores between MG and HTG. HHGS scores in HG, HTG, and CTG were significantly lower than that in MG, in which the HHGS score in CTG was significantly lower than those in HG and HTG. These results were consistent with previous observations and semi-quantitatively indicated the enhanced cartilage repairing effect of the HA and hAMSCs combination.

### The Regulation of Regenerative Microenvironment

**FIGURE 7:**
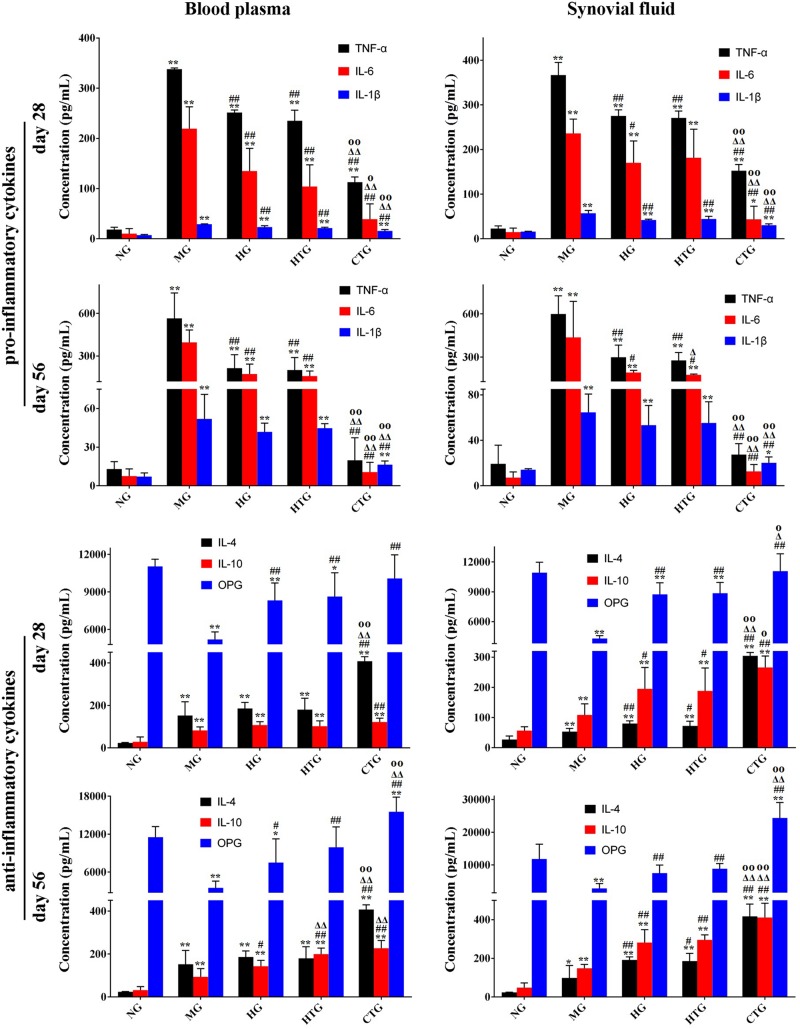
Expression of cytokines in blood plasma and synovial fluid in rat OA models. NG, LG-DMEM; MG, Model group; HG, 0.05 mg/mL HA; HTG, hAMSCs 1×10^6^; CTG, HA (0.05 mg/mL) + hAMSCs (1×10^6^). The data are expressed as mean ± sd (*n* = 6), *P < 0.05, **P < 0.01 vs. NG; ^#^*P* < 0.05, ^##^*P* < 0.01 vs. MG; ^Δ^ *P* < 0.05, ^Δ^ ^Δ^ *P* < 0.01 vs. HG; ^O^*P* < 0.05, ^OO^*P* < 0.01 vs. HTG.

Next, we tested the secretion of cytokines that regulated regeneration in the knee joint synovial fluid and the blood plasma. The linear correlation coefficient (*R*^2^) of standard curves for all cytokines was greater than 0.99, showing excellent linearity (Supplementary Figure S5). After 28 days, ELISA analysis in both the synovial fluid and blood plasma illustrated that compared with the MG group, anti-regenerative factors (TNF-α, IL-6, and IL-1β, all of which were pro-inflammatory cytokines) were significantly down-regulated, while pro-regenerative factors (IL-4 and IL-10, both of which were anti-inflammatory cytokines, and OPG, which was a protective cytokine for bone tissues) were significantly up-regulated in the HG, HTG, and CTG groups (Figure 7); the secretion of all cytokines in HG and HTG groups showed comparable level without significance, while that in the CTG group was significantly decreased for anti-regenerative factors and significantly increased for pro-regenerative factors, even reaching comparable or very close levels for anti-regenerative factors and significantly higher levels for pro-regenerative factors at the end of the treatment (Figure 7). From these results, it could be seen that the use of HA alone, hAMSCs alone, and the combination of both could regulate the expression of inflammatory factors, reduce the inflammatory response, and promote the secretion of OPG, in which the synergistic regulation effect of the combination treatment group was the most obvious.

### Homing and Colonization of hAMSCs-Derived Cells and Expression of Gene *Col2*α*1* in Cartilage Tissues

**FIGURE 8:**
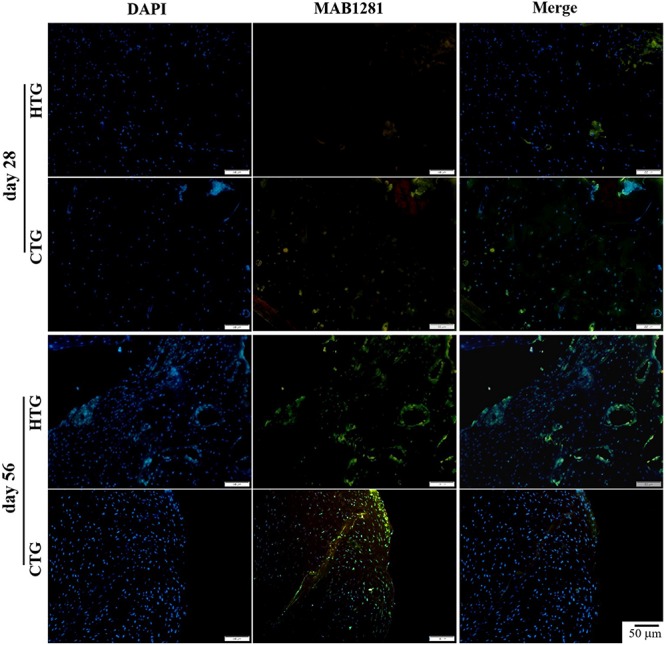
Trace of hAMSCs by MAB1281 antibody in rat OA models. MAB1281 positive cells (green) indicate the human-derived cells. Nucleus: DAPI (blue). NG, LG-DMEM; MG, Model group; HG, 0.05 mg/mL HA; HTG, hAMSCs 1×10^6^; CTG, HA (0.05 mg/mL) + hAMSCs (1×10^6^). Scale bar: 50 μm. This figure shows the representative immunofluorescence stainings of each group containing three rats.

To determine the distribution of hAMSCs-derived cells in a rat OA model, we performed immunofluorescent staining using the MAB1281 antibody in both HTG and CTG groups after the transplantation. At days 28 and 56, compared with the CTG group, MAB1281 positive cells were fewer than in the HTG group, indicating that co-treated HA contributed to the survival and colonization of injected hAMSC (at least in part) in the OA model (Figure 8). We also evaluated the expression of gene *Col2*α*1* in knee joint cartilage, which encoded the alpha-1 chain of collagen II. The expression of gene *Col2*α*1* was much higher in the CTG group, which was significantly higher than the MG group, about 3-fold higher than the HTG group, and even approximate to the normal group (Supplementary Figure S6). These results suggested that if combined with HA, the synthesis of collagen II in the regenerated cartilage was enhanced.

## Discussion

Due to its high incidence, OA has become the second global leading cause for work disability in recent decades. Its clinical prognosis, however, is unsatisfactory at present, and existing treatments are not performed in a simple enough manner. It is thereby urgent to establish an effective and simple remedy to treat this disease in clinic. The development of regenerative medicine brings us stem cell-based injection therapy. On the basis of the excellent properties of hAMSCs and HA, we infer that their combination injection holds high application potential.

Interestingly, in the rodent MIA-induced OA model established in the present study, we observed considerable regeneration of cartilage tissues in the combination treatment group (CTG);the curative effect in the CTG group was much better than that in the OA model rats only injected with HA (HG) or hAMSCs (HTG). The repaired cartilage was quite similar to the normal cartilage in terms of gross morphology, MRI imaging, tissue composition, and matrix expression, which illustrates favorable regeneration degree and function. If the OA was left untreated, the cartilage would deteriorate as shown in the MG group. Although a little bit better than the MG group, the repair efficacy of the HG and HTG groups was still weak, much less than the CTG group. Hence, the synergistic repairing effect of HA and hAMSCs was obvious, both of which were organic components of this therapy. We later demonstrated that the effect was derived from the superior properties of both HA and hAMSCs as well as their interaction.

Based on its excellent physicochemical properties, HA has been widely used as a scaffold material in the field of articular cartilage repair. HA can be used as a carrier to provide superior adhesion sites for mesenchymal stem cells (MSCs), enabling them to proliferate, differentiate, and migrate better, and to repair damaged cartilage (Park et al., 2015). In addition, the cartilage-like scaffold material made of HA is more conducive to the promotion of human BMSCs to repair the osteoarthritis in cartilage explant model and differentiation into chondrocytes *in vitro* (Kon et al., 2015; Muiños-López et al., 2017). As HA is a crucial constitute in knee cartilage, we infer that the presence of exogenous HA can reduce the catalysis of HA in tissues and cooperate into the tissue if necessary, and a proper concentration of exogenous HA should facilitate the dynamic equilibrium of structural HA in the cartilage. The positive effects of HA can also be proved by the hAMSC trace experiment by both MRI and immunofluorescent staining. Compared with the HTG group, hAMSCs colonized far better in the CTG group. The colonized hAMSCs survived and incorporated the tissue regeneration well. The neo-tissue secreted large amounts of collagen II, suggesting that the tissue is performing its normal function and the hAMSCs have been most likely differentiated into chondrocytes. The inflammation was also suppressed obviously. It is apparent that the enhanced colonization of hAMSCs in damaged tissue is one of the important reasons for the excellent repair efficacy.

The effect of using HA alone on cartilage repair is debated at present. It has been confirmed that the use of HA alone has obvious curative effects for the cartilage injury and has been widely used clinically (Sampson et al., 2013; Park et al., 2015). HA is commonly used in the treatment of cartilage damage in clinic as a second-generation HA joint cavity injection product (Vincent, 2019). However, there are also some reports showing its very limited efficacy (Rutjes et al., 2012; Jevsevar et al., 2015). In the present study, we found that HA alone showed positive but weak repairing effect on cartilage injury site, where joint effusion still existed, chondrocyte arrangement was disordered, cartilage matrix GAG was lightly stained, and collagen II was weakly positively expressed. This might also be associated with the low concentration of HA used in this study (0.05 mg/mL). Therefore, the effectiveness of HA alone in the treatment of hAMSCs still requires further evaluation.

On the other hand, the efficacy of using MSCs alone on cartilage repair is also controversial. There are studies reporting that the use of MSCs alone to treat cartilage injury has very limited effect (Dhinsa and Adesida, 2012). In contrast, other studies have shown that MSCs play an important role in relieving pain, delaying the onset of arthritis, and repairing cartilage damage (Dhinsa and Adesida, 2012; Rameshbabu et al., 2016). In the present study, we found that compared with the MG group, hAMSCs alone had a certain ability to repair cartilage damage in HTG group, comparable to that of using HA alone in the HG group. However, when HA and hAMSCs were combined together, the therapeutic effect was greatly improved, and the damaged cartilage, after 56 days of treatment, was even restored close to the level of intact cartilages. The pro-regeneration effect of HA on MSCs in cartilage tissue repair has also been demonstrated by other researchers (Lee et al., 2007; Ha et al., 2015). In the present study, because HA alone has weak repairing efficacy, we believe that hAMSCs are indispensable in a better therapy, which provide seed cells, and HA is an assistant but also indispensable reagent facilitating hAMSCs activities.

Inflammatory factors are crucial regulators for neo-tissue formation. Typically, pro-inflammatory factors (e.g., TNF-α, IL-1β, and IL-6 in this study) are harmful to tissue remodeling, but anti-inflammatory factors (e.g., IL-4 and IL-10 in this study) are beneficial (Goldring et al., 1988; Guerne et al., 1990; Dingle et al., 1991; Dechanet et al., 1994; Bone, 1996; Goldring, 2000; Dai et al., 2018). Intriguingly, in this study, compared with the MG group, the expression levels of these factors in HG, HTG, and CTG in blood plasma and synovial fluid were all significantly down-regulated, and the down-regulation extent in the CTG group was significantly stronger than that in the HG and HTG groups. These results indicate that HA alone and hAMSCs alone both reduce the release of pro-inflammatory factors, while their combination further synergistically enhances the reduction of the release. Further, anti-inflammatory factors IL-4 and IL-10 can also be regulated. In this study, the expression level of IL-4 and IL-10 in plasma and synovial were up-regulated in the HG, HTG, and CTG groups in blood plasma and synovial fluid, and the up-regulation extent was significantly stronger in the CTG group than that in the HG and HTG groups. These results indicated that HA alone and hAMSCs alone both increased the release of anti-inflammatory factors, while the synergetic effect of their combination was obvious. Taken together, the repair effect in the CTG group may be due in part to the enhanced immunomodulatory effects of the hAMSCs/HA cocktail and/or tissue response induced by the cocktail injection, thereby protecting the articular cartilage from further damage.

We also evaluated the expression of another pro-regenerative cytokine, osteoprotegerin (OPG), in plasma and synovial fluid. OPG, a member of the tumor necrosis factor (TNF) receptor superfamily, is secreted by osteoblasts. OPG blocks receptor activator of nuclear factor κB ligand (RANKL) and nuclear factor κB receptor activating factor (receptor activator of NF-κB, RANK) by competitively binding to RANKL binding, and in turn, inhibits the differentiation and maturation of osteoclast and induces apoptosis in osteoclasts (Krieger and Odrowaz-Sypniewska, 2004). This study found that the expression of OPG in plasma and synovial fluid held the same trend as anti-inflammatory cytokines, again indicating the positive effects of HA alone and hAMSCs alone, and the synergetic effect of the combination of HA and hAMSCs. The result also implicated that the cartilage repair might involve the inhibition of osteoclast activity by OPG, which further protected bone and cartilage, and also that the repair of cartilage was relevant to the healthy state of the sub-chondral bone and cells inside it. Hence, taking the cartilage and sub-chondral bone (maybe also synovium) as a whole may be a next generation therapeutic paradigm for joint cartilage regeneration.

## Conclusion

Overall, we report, for the first time, that intra-articular injection of the cocktail (100 μL) of HA (300 kDa, 0.05 mg/mL) and hAMSCs (1×10^6^) can strongly regenerate damaged knee cartilage in an MIA-induced rat OA model. The therapeutic effect can be attributed to the following reasons: (i) hAMSCs are an excellent stem cell source for cartilage regeneration with potent capabilities in terms of proliferation, differentiation, modulation of the cytokine secretion, and integrity into host tissues; (ii) HA is a regeneration-friendly exogenous reagent with the merits of lubrication, protection, cytokine modulation, and environmental refinement functions; and (iii) HA can synergistically and comprehensively enhance the activities of hAMSCs in cartilage repair, including proliferation, differentiation, cytokine modulation, colonization, and matrix formation. This therapy also has several attractive features: (i) the knee joint injection treatment is effective yet simple; (ii) HA is an FDA-approved, safe, clinically used, and highly biocompatible reagent; (iii) hAMSCs are easy to obtain, abundant in source, free of ethical restriction, non-tumorigenic, and capable of immunomodulation; and (iv) hAMSCs are immunoprivileged, which can be well-embedded in allogenic human hosts and serve other individuals. We therefore believe this cocktail is a promising solution to articular tissue degeneration with the potential to realize rapid clinic translation.

## Data Availability Statement

The datasets used and/or analyzed in this work are available from the corresponding author upon reasonable request.

## Ethics Statement

The use of human amnion and the procedure of animal trials were approved by the Ethical Committee of the Affiliated Hospital of Zunyi Medical University. Informed consent was acquired from the donor prior to the amnion collection in accordance with the Declaration of Helsinki.

## Author Contributions

J-HX: conceptualization and funding acquisition. A-TW, Q-FZ, and Y-JZ: investigation and methodology. J-HX and C-YY: supervision. A-TW, Q-FZ, and N-XW draft writing. N-XW, J-HX, and C-YY: manuscript revision. R-ML and YL: constructive discussion. All authors performed the manuscript checking and correction.

## Conflict of Interest

The authors declare that the research was conducted in the absence of any commercial or financial relationships that could be construed as a potential conflict of interest.
