# Supplementary material for: Cocktail of Hyaluronic Acid and Human Amniotic Mesenchymal Cells Effectively Repairs Cartilage Injuries in Sodium Iodoacetate-Induced Osteoarthritis Rats
Source: Front Bioeng Biotechnol. 2020 Mar 6;8:87. doi: 10.3389/fbioe.2020.00087 (PMC7068044; doi:10.3389/fbioe.2020.00087)
Supplement: Supplementary file 1 [file Data_Sheet_1.pdf]

Supplementary Material

Supplementary Figures

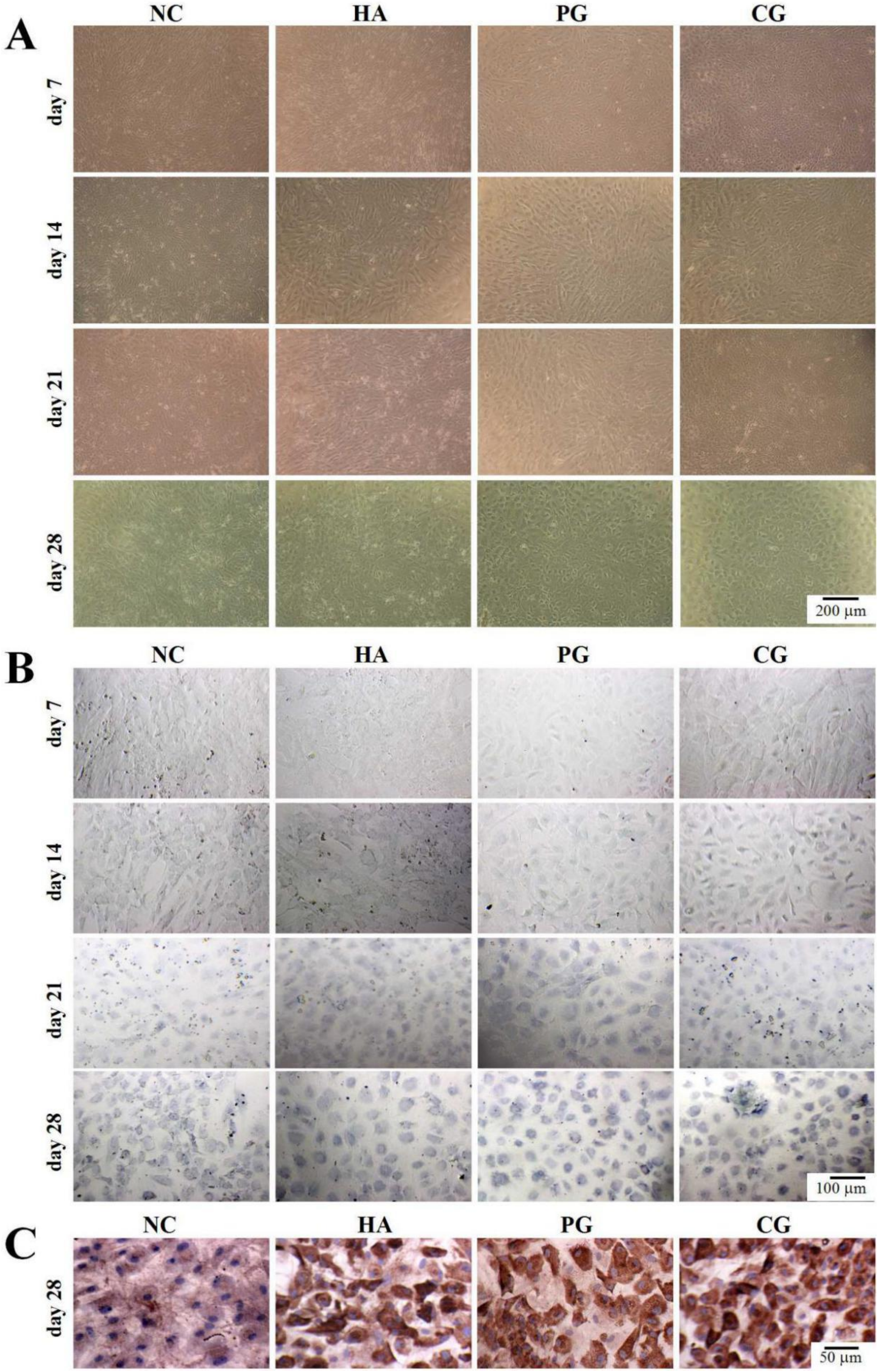
A
NC
HA
PG
CG
day 7
day 14
day 21
day 28
200 μm
B
NC
HA
PG
CG
day 7
day 14
day 21
day 28
100 μm
C
NC
HA
PG
CG
day 28
50 μm

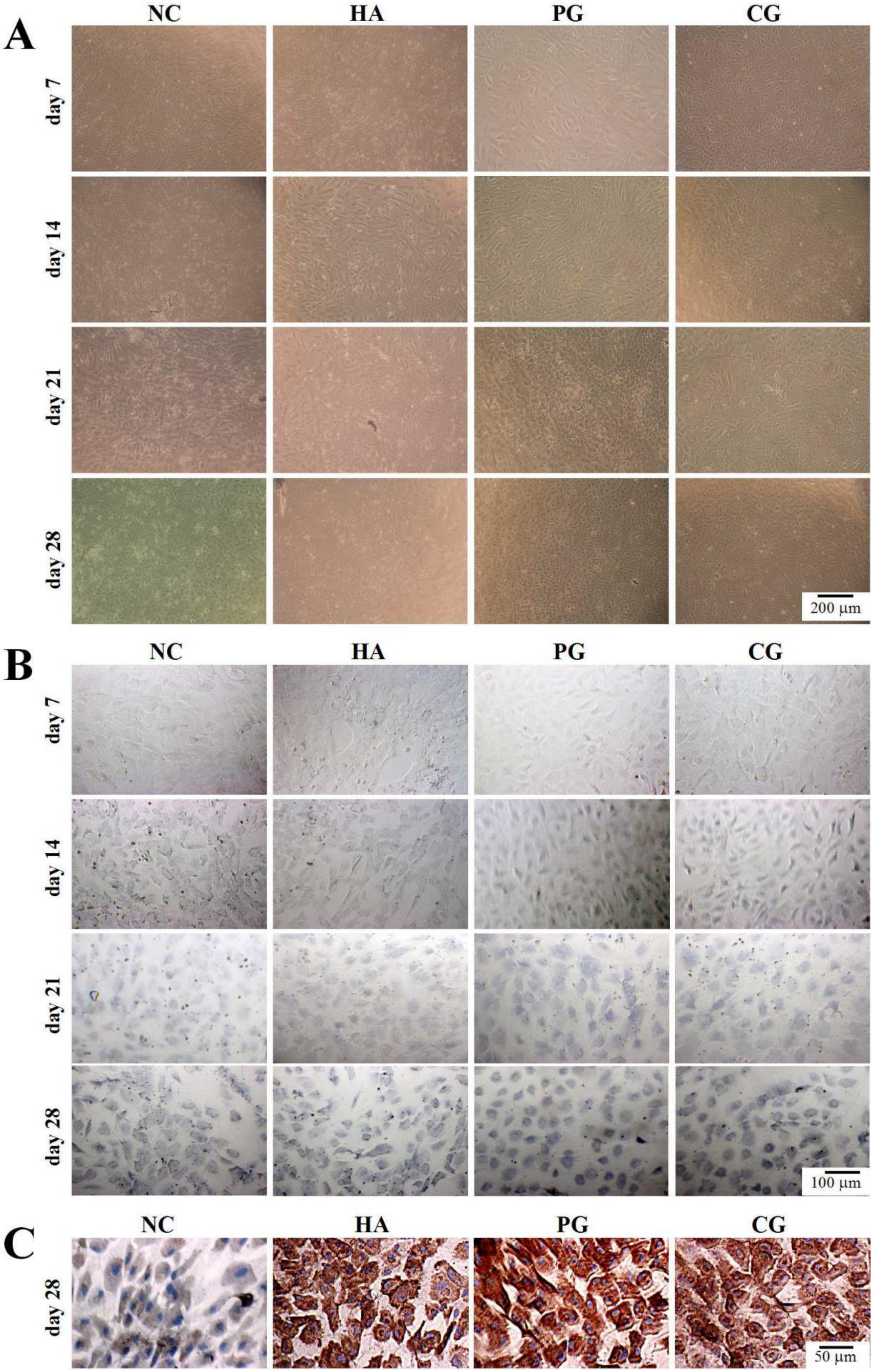

Figure S1 The other two repeats of chondrogenic differentiation of hAMSCs in vitro

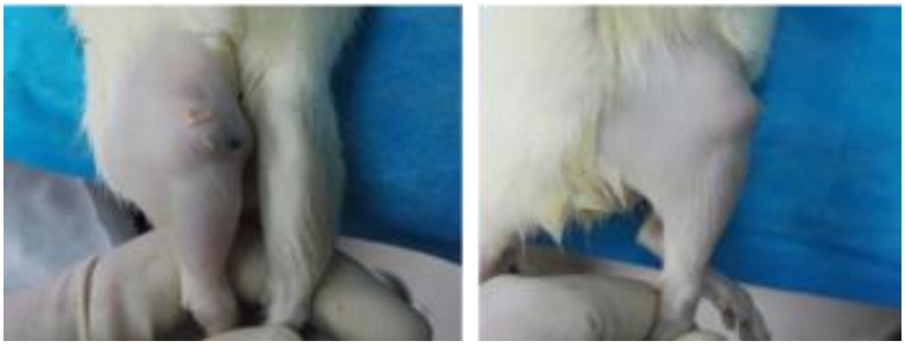

Figure S2 The swollen knee joint of rats after OA induction. Left: top view, Right: side view.

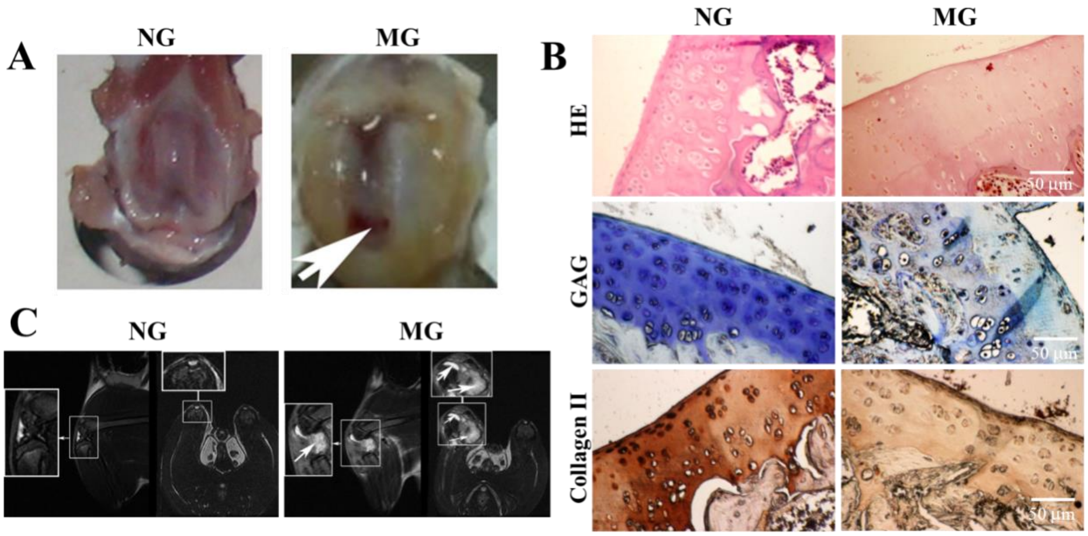

Figure S3 Establishment of the OA model. (A) Explanted tissue. (B) Histological staining. Top: HE staining; Middle: Tolune blue O staining for GAG; Bottom: Immunohistochemical staining for Collagen II. (C) MRI. NG, normal group; MG, model group. In MG group, the arrows indicate the high signal of MRI showing the cartilage injury/inflammation.

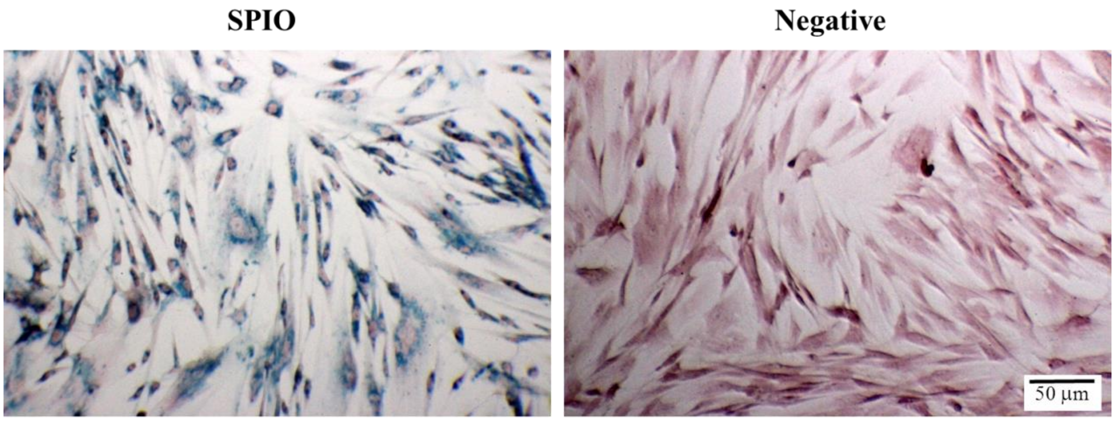

Figure S4 Detection of the labeling rate of hAMSCs by SPIO using Prussian blue staining.

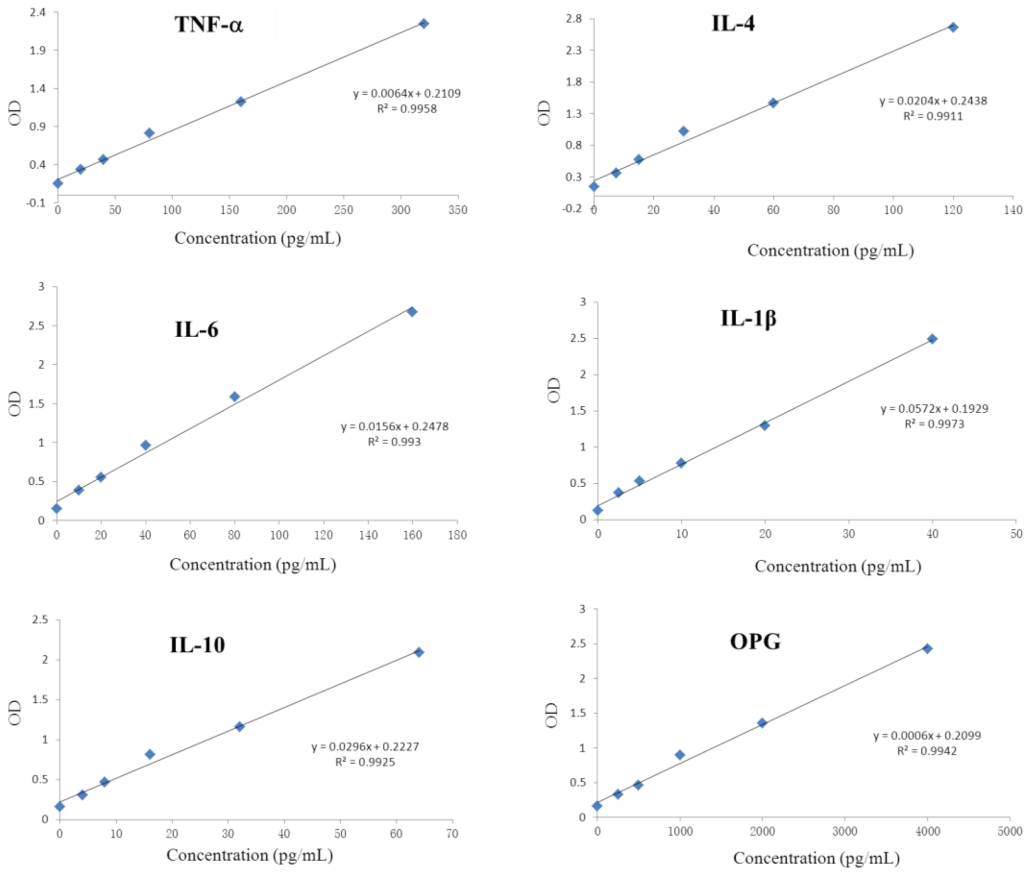

Figure S5 Standard curves of cytokines

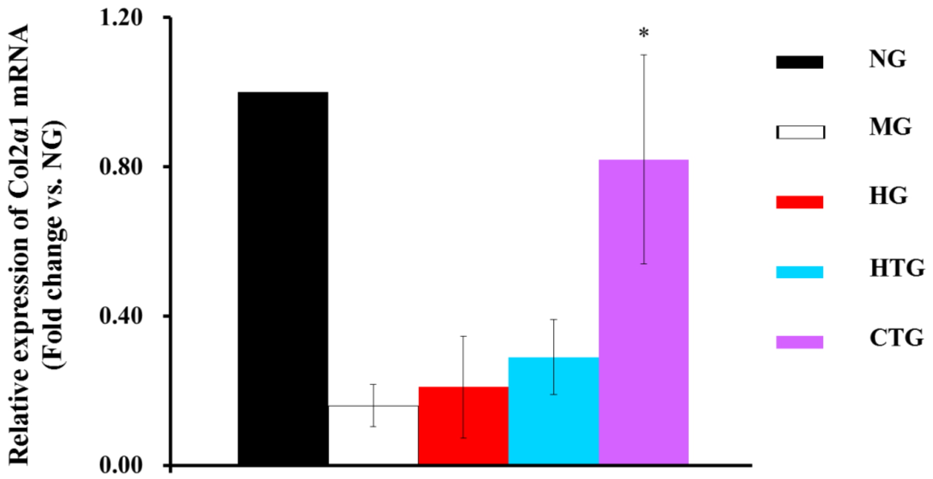

Figure S6 Relative expression of collagen II gene *Col2α1* after 56 days of treatment. NG, LG-DMEM; MG, Model group; HG, 0.05 mg/mL HA; HTG, hAMSCs 1×10^6; CTG, HA (0.05 mg/mL)+hAMSCs (1×10^6). The data are expressed as mean±sd (n=3), $^*P$<0.05 *vs.* MG, HG, and HTG.
